# Laparoscopic resection of a primary hydatid cyst of the adrenal gland: a case report

**DOI:** 10.1186/1752-1947-1-61

**Published:** 2007-08-06

**Authors:** Gianlorenzo Dionigi, Gianpaolo Carrafiello, Chiara Recaldini, Fausto Sessa, Luigi Boni, Francesca Rovera, Renzo Dionigi

**Affiliations:** 1 Department of Surgical Sciences, University of Insubria, Varese, Italy; 2 Department of Radiology, University of Insubria, Varese, Italy; 3 Department of Human Morphology, University of Insubria, Varese, Italy

## Abstract

**Background:**

Echinococcosis rates vary in different parts of the world. Italy is regarded as a middle to high risk country with over 1,000 cases requiring surgery each year. Liver (45–75%) and lung (10–50%) are the most frequent locations of this parasitosis.

**Case presentation:**

The authors report a clinical case of a 62 year old woman, admitted to hospital with left flank pain. Plain radiographs of the abdomen, ultrasound, CT and MRI scans were performed and the presence of a 3-cm lesion of the left adrenal gland was demonstrated. A diagnosis of hydatid cyst was made. The patient underwent transabdominal laparoscopic left adrenalectomy. Histopathological examination confirmed the presence of a hydatid cyst in the left adrenal gland.

**Conclusion:**

A hydatid cyst was correctly diagnosed on the basis of radiologic findings. The uncomplicated cyst was successfully resected using a laparoscopic approach. The pathological features of this case are presented in this paper.

## Background

Echinococcosis in humans is a zoonotic infection caused by larval stages of cestode species of the Echinococcus genus [1]. The cystic form is caused by Echinococcus granulosus. The liver is the most frequent organ involved (45–75%), followed by the lung (10–50%) [1]. Heart, spleen, kidney and brain are less commonly involved representing about 10% of the total number of cases [1]. Hydatid cyst of the adrenal gland constitutes less than 1% of all cases [2-6].

Echinococcosis occurs mostly in the northern hemisphere, in geographically limited foci of Europe, Turkey, many areas of the former Soviet Union, Iran, Iraq, China, and Japan [1,7]. Surveillance for echinococcosis in Europe has revealed an incidence of 1–20 cases per 100,000 persons per year [1,7]. Echinococcosis seems to be still endemic in Italy where there are less than 1,000 surgical procedures each year [1,7]. Data from epidemiological studies in Italy show that this parasitosis is often a work-related disease [1,7]. Dairy farming seems to be a risk factor. 60% of patients are involved in vocational or part-time farming, gardening, forestry, or hunting [1,7]. Humans may become infected with Echinococcus granulosus by eating contaminated food or by direct contact with infected animals. Mass screenings have identified symptomatic and asymptomatic infections in patients ranging in age from 6 years to the very elderly. About 80% of patients have symptoms, but small (<5 cm in diameter) and uncomplicated cyst are usually asymptomatic. In endemic areas, the male-to-female ratio is approximately equal [1,7].

We report a case of a symptomatic patient with an Echinococcus cyst of the left adrenal gland. Initially observed on a plain abdominal x-ray, the cyst was correctly diagnosed on the basis of computer tomography (CT) scan images and magnetic resonance imaging (MRI) findings and confirmed at surgery. The pathological features of this case are also presented in this paper.

## Case presentation

**Figure 1:**
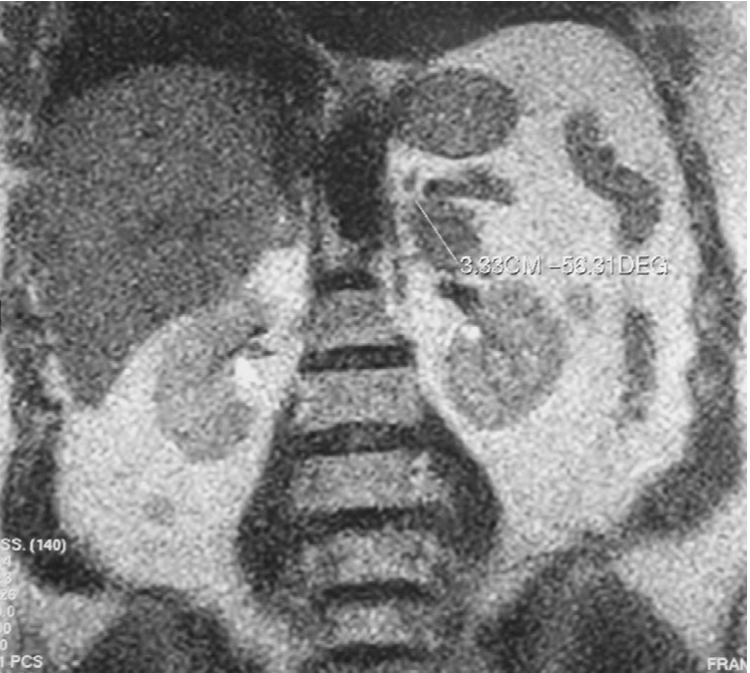
MRI T2-weighted imaging: central low-signal-intensity due to the extinguished cystic membrane and a more hypointensive rim of the cyst due to wall calcification.

**Figure 2:**
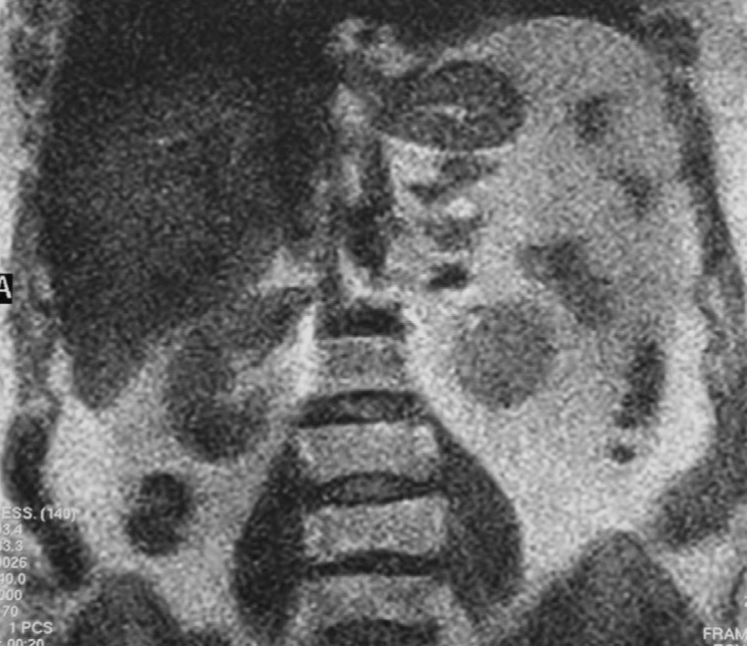
MRI T2-weighted imaging: little hyperintensive nodular lesion in the upper pole of the cystic lesion related to a daughter cyst.

**Figure 3:**
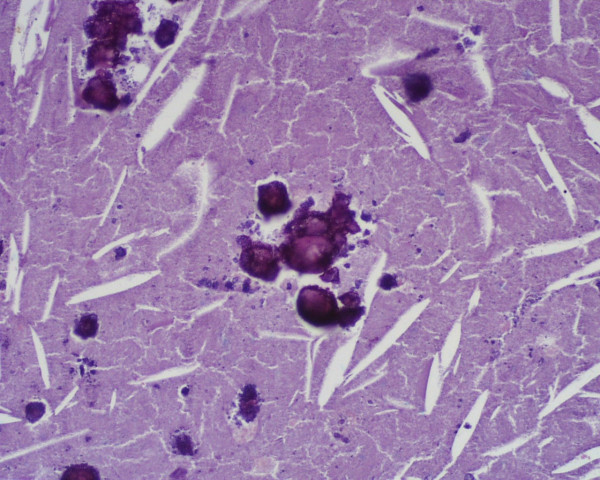
Histology (stain hematoxylin and eosin; magnification ×200): necrotic material with calcified protoscolic.

A 62-year-old woman was referred to the Department of Surgical Sciences, University of Insubria, Varese, for left flank pain. Physical examination was unremarkable. Complete blood cell count, electrolytes, eosinophil count, serum biochemistry and urinalysis were within normal limits. Plain radiographs of the abdomen showed a small round opacity, about 3-cm in diameter, in the left upper region of the abdomen, with evidence of calcifications. Chest X-ray was normal. The patient underwent ultrasound examination (US) that showed a calcified heterogeneous cystic mass between the superior left kidney pole and spleen in the retroperitoneal area. CT scanning demonstrated the presence of a solitary cystic mass with a calcified wall within the left adrenal gland with no enhancement after iv contrast media injection. No other intra-abdominal or intra-thoracic masses were found, either in the liver, peritoneum or lung. MRI T2-weighted imaging showed a hypointensive lesion with a calcified wall, and a peripheral hyperintensive nodule (Figure 1, 2). CT and MRI findings suggested adrenal echinococcosis. The specific serology for immunoglobulin anti-E. granulosus resulted positive 1:61 (n.v. < 50). Urinary catecholamines and metanephrine levels were within normal limits. The patient confirmed that she had had animal contact. The albendazole preoperative therapy resulted in the disappareance of pain. The diagnosis was confirmed by surgery. The cyst was removed by a transabdominal laparoscopic approach. The exploration of the rest of the peritoneal cavity did not reveal any other lesions. The area around the cyst was carefully packed with gauze soaked in 20% hypertonic saline solution. The cyst was not drained nor aspirated during the procedure. An appropriate dissection plane between the cyst and the adrenal gland could not be found as it was hard and adhesive, so both the cyst and the left adrenal gland were endoscopically removed. Pathological examination of the specimen confirmed that it was a hydatid cyst of the adrenal gland. Macroscopic pathologic examination showed a circumscribed unilocular cystic lesion 3-cm in diameter with a thick fibrous wall of 2-mm. It weighed about 57 grams. On palpation there were stone hard areas of calcification within the cyst wall. Examination of the contents of the cyst showed the typical clear fluid of a hydatid cyst with sand pasty material and calcified bodies. Moreover necrotic material with protoscolices was present (Figure 3). No protein was detected and the glucose level was 72 mg/dl. The patient's postoperative course was uneventful. The patient was discharged on the third postoperative day. Therapy with albendazole was administrated for the 4 weeks following surgery (30 mg/kg/day orally). Six months later, the patient is well and in good condition with no further symptoms.

## Discussion

This case is unusual due to the organ involved. Hydatid cysts can occur almost anywhere in the body [1,7]. The adult worm lives in the small intestine of the primary host. Ova are passed in feces and ingested by the intermediate host, which may include humans. Hatched embryos migrate through the intestinal mucosa, enter venules and lymphatics, and reach the liver. If embryos bypass the liver, they can reach the lungs via the inferior vena cava and heart. Embryos may reach other organs as the adrenal glands via the systemic circulation. A hydatid cyst of the adrenal gland is extremely rare: only 15 cases have been described to date in the English language medical literature with an incidence of 0.5% [2-6]. Hydatid cysts in organs other than liver or lungs are usually part of generalized echinococcosis, and only rarely are they primary cysts as described in this case. Cysts of the adrenal gland are usually unilateral (90%) and show no special predilection for either side [2-6].

The growth of a hydatid cyst is usually slow and asymptomatic. In this case, the patient had nonspecific symptoms. The expansion of a larger cyst, a number of cysts or inflammatory reaction around a cyst with irritation of the adjacent peritoneum may cause pain in the flank. Occasionally, a palpable mass may be found [2-6]. The major complication risk is rupture of a hydatid cyst of the adrenal gland into the peritoneum or retroperitoneum, which may trigger anaphylactic shock. Other complications can include local infection, fistula, hemorrhage, or compression of adjacent tissues [2-6]. Rarely an adrenal hydatid can cause arterial hypertension which has been described as the Goldblatt phenomenon [4].

A variety of tumors can be located in the adrenal gland and must be considered in the differential diagnosis [8]. Cysts of the adrenal gland are rare, but with the wider application of US and CT more adrenal cysts are being detected incidentally [8,9]. The aetiological morphological classification of adrenal cyst is as follows: (1) non-neoplastic cysts and (2) neoplastic cysts. Non-neoplastic cysts can be: (a) endothelial cyst (with an incidence of 45%), (b) pseudocyst (39%), (c) epithelial cyst (9%), and parasitic cyst 7% (generally echinococcal) [8]. The incidence of adrenal cysts in the general population is about 0.06% [8]. In endemic regions hydatid cysts constitute most causes of adrenal cysts requiring surgery [8]. As Italy is a endemic area of hydatid disease, the hydatidic etiology of this patient's cyst was presumed preoperatively [8]. Diagnosis is easy with good imaging technique; US, CT and MRI are suitable also for post-treatment follow-up [2-6,9]. Radiologic findings can range from cystic lesions to a completely solid appearance according to the stage of growth of the cyst or associated complications [9]. US is particularly useful for the detection of septa and hydatid sand with floating echinococcal membranes [9]. In 2003, the World Health Organization proposed a classification based on US features and included five types: type 1 is a well-defined, anechoic lesion; type 2 demonstrates separation of the membrane (the "water lily" sign formed by the undulating membrane); type 3 is characterized by the presence of septa and intraluminal daughter cysts. Type 4 is a nonspecific solid mass. Type 5 is a solid mass with a calcified capsule [9]. The case described in this report is type 5. However, US cannot differentiate hydatid cysts from other adrenal cysts. CT scanning may reveal fluid content within the cyst, with a density close to that of water; daughter cysts when present appear as curved septations [10]. On CT the cyst walls can range in thickness from 2-mm to 1-cm, with the wall representing the combined pericyst, ectocyst, and endocyst [10]. A hydatid cyst may be very large: cysts of 10–20 cm have been reported [2-6]. The rate of growth may be fairly rapid, with doubling times of less than 6 months [2-6]. MR and CT images will show the exact anatomic extent, size, volume and position of the mass, the number of cysts, the relationship to other organs and possible complications. Few reports of MRI images of adrenal hydatid disease have been published. Authors report that MRI in these cases is more specific than CT [10]. On MRI the complex cyst contents can be well displayed, and the cyst membrane, whether collapsed or not, can be clearly seen as a low-intensity curvilinear structure on both short and long TR spin-echo images [10]. The mass in our case did not have the characteristic signal intensity of a cystic lesion, but a central low-signal-intensity on T2-weighted MRI due to the extinguished cystic membrane and a more hypointensive rim of the cyst due to wall calcification. Moreover a little hyperintensive nodular lesion was found in the upper pole of the cystic nodule probably related to daughter cysts. Pedrosa recently reported that daughter cysts when present are seen as cystic structures attached to the germinal layer and that they are hypointense relative to the intracystic fluid on T1-weighted images and hyperintense on T2-weighted images [10].

Serological tests are based on the reaction and precipitation between the test antigen and the circulating antibodies in the host [1-7]. The sensitivity and specificity of available tests depend on the quantity of antigens. Serological test use partially purified hydatid antigen or antigen 5 [1,7]. Complement fixation, hemogglutination, latex agglutination, and bentonite flocculation test are also available [7]. Complete blood cell count was within normal limits in this patient: in a recent paper, eosinophilia (more than 5% eosinophils) occurred in 40% of patients in a reported series [7]. In fact, eosinophilia is believed to be more likely to occur if the cyst leaks [7].

Antihelmintic agents have been used in the treatment of systemic echinoccosis in endemic areas [7]. There are reports that antihelmintic agents can reduce the size of cysts in some cases, however the results are not satisfactory and this treatment should be limited for disseminated and recurrent cysts or in cases where surgery is contraindicated [7]. In this case, antiparasitic agents were used prior to surgery and after surgery to prevent further implants and secondary hydatid seeding. Puncture was contraindicated because of potential complications such as anaphylactic shock and spread of daughter cysts [7].

Surgery remains the mainstay for the treatment of hydatid cysts [2-6].

The rapid development of laparoscopic techniques has encouraged surgeons to replicate principles of conventional hydatid surgery using a minimally invasive approach. This case reports the use of a transabdominal laparoscopic adrenalectomy. The benefits of endoscopic surgery compared to open traditional surgery are well known and include less surgical scarring, less operative pain and shorter length of postoperative stay [6]. In general, drawbacks of this technique are the fact that a minority of patients qualify for this approach as the volume of the adrenal masses to be removed often exceeds the possibilities of endoscopic surgery. The learning curve is quite long for the surgeon and the availability of surgeons experienced both in endocrine and in endoscopic surgery is low. Uncomplicated cysts can be treated successfully through a laparoscopic approach, but adhesion to vital structures can sometimes make this impossible [6]. With proper patient selection, several reports confirmed the feasibility of laparoscopic hydatid surgery. In fact, laparoscopic adrenalectomy offers superior visualization and access to the adrenal gland and avoids the major laparotomy incision necessary in open procedures, and it has the advantage of allowing the surgeon to explore the peritoneal cavity [6].

## Competing interests

The author(s) declare that they have no competing interests.

## Authors' contributions

GD acquisition of data

FS, LB, FR, GC, CR: drafting of manuscript

CR critical revision and supervision
